# A Fully-Printed CRLH Dual-Band Dipole Antenna Fed by a Compact CRLH Dual-Band Balun

**DOI:** 10.3390/s20174991

**Published:** 2020-09-03

**Authors:** Muhammad Kamran Khattak, Changhyeong Lee, Heejun Park, Sungtek Kahng

**Affiliations:** Department of Information & Telecommunication Engineering, Incheon National University, Incheon 22012, Korea; Kamrankhattak01@gmail.com (M.K.K.); Antman@inu.ac.kr (C.L.); h.park.inu@gmail.com (H.P.)

**Keywords:** dual-band dipole, CRLH antenna, dual-band balun, CRLH balun, wireless communication

## Abstract

In this paper, a new design method is proposed for a planar and compact dual-band dipole antenna. The dipole antenna has arms as a hybrid CRLH (Composite right- and left-handed) transmission-line comprising distributed and lumped elements for the dual-band function. The two arms are fed by the outputs of a compact and printed CRLH dual-band balun which consists of a CRLH hybrid coupler and an additional CRLH phase-shifter. Its operational frequencies are 2.4 and 5.2 GHz as popular mobile applications. Verifying the method, the circuit approach, EM (Electromagnetics) simulation and measurement are conducted and their results turn out to agree well with each other. Additionally, the CRLH property is shown with the dispersion diagram and the effective size-reduction is mentioned.

## 1. Introduction

Nowadays, wireless connectivity from equipment to other equipment and technological convergence becomes more vibrant. This results from a need to push the current limits. For instance, the 2.4 GHz band in the WLAN (Wireless local area network) frequency is commonly used, but this band is not sufficient to provide proper amounts of data because of excessive use and interference with other wireless communication methods using the same frequency band (e.g., Bluetooth, DCP, and ZigBee). So, to meet the frequency requirements, a number of multiband antennas have been proposed with various structures such as IFAs (Inverted-F antennas), bow-ties, slots and monopoles [1,2,3,4]. Pushpakaran et al. proposed a dog bone shape dual-band dipole antenna for WLAN applications. They presented a method for achieving a dual-band property by using the stacking technique [5]. Deepak [6] proposed a dipole antenna along with a folded element for multi-band operation. To eliminate the return current leak of the SMA (Subminiature version A) connector, they separate the dipole arms by using the double sides. Sim et al. reported multi-band asymmetric dipole antenna for WLAN operation. For the feeding of dipole antenna, they are using the 50-ohm coaxial cable as positive and negative on the two arms [7]. J. Huang used a tapered transition the balance feed line in [8]. The trapezoid dipole arms are printed on one side of the substrate and the other single dipole is formed on the opposite side. Nair [9] presented an F-shaped slot line to feed a dual-band dipole antenna which are fed by an SMA connector. H. Azeez modified a dipole with a pair of E-shaped conductive arms to generate multiple resonance [10]. Its feeding scheme is the same as [9]. Others put their radiating elements near a large metal ground like Alekseytsev who coupled a slit with an overpass metal-line to excite an open loop and a parasitic for two resonance frequencies [11]. More complicated configurations are built by Barani, such as one PIFA (Planar Inverted-F antenna) conjoined with PIFAs, with monopoles and slits on the ground to increase the number of bands [12]. Because the ground-edge mounted antennas occupy a large footprint, multi-band antennas are made as 3D structures. Tang adopted vertical loops passing through via-holes of two parallel lines [13]. Sreelakshmy put a thick disk on a vertical feed-line, and formed two asymmetric holes splitting one angular and radial mode into two, and used them for the dual-band characteristics [14].

The previously mentioned multi-band dipole antennas adopt SMA and coaxial feeds as an unbalanced signal port. To cope with that, the balanced to unbalanced (balun) structure is needed for a dipole antenna. Besides, to feed the dual-band dipole antenna, it is necessary to combine the dual-band balun. Liu et al. proposed the CRLH (Composite right- and left-handed) transmission line balun, in which they made the virtual ground for the odd mode and shunt inductor to remove a via. So, the phase difference between the two output ports is around 180° at 1.5 and 3.6 GHz [15]. Tseng et al. developed a CRLH balun to control the phase slope. As a result, they obtained the multi-band property by using the two branches where one arm consists of the RH-TL (Right-handed transmission line) and the other part is based on the LH-TL (Left-handed transmission line) with lumped elements [16]. According to odd-mode and even-mode analysis, the dual-band baluns are proposed with open-stub and BPF (Bandpass filter) [17,18]. Huang et al. reported a microstrip-based Marchand-type balun. They could achieve the high selectivity by using the roll-off of the filter [19]. Isolation between two bands can be better by making an SIR (Step impedance resonator) feedback loop between the two output branches by Li [20]. Multiple loops causing the phase difference are formed through layers with vertical lines as bridges [21]. Kahng et al. proposed a CRLH-based balun for common-mode current indicator which is employed in the chip and PCB EMC (Printed circuit board electromagnetics compatibility) problems. The balun consists of the branch-line coupler and the compact metamaterial phase shifters that are horizontally cascaded [22]. It works for a single band.

In this paper, a compact and fully printed metamaterial dual-band balun and metamaterial dual-band dipole antenna are proposed and put into one structure. The dual-band balun has the CRLH hybrid branch line-coupler and a CRLH phase-shifter. The conventional coupler and phase shift lines are replaced by miniaturized phase-shift lines having +90° for one frequency and −90° for another frequency for dual-band and low-cost fabrication. Instead of power-divider shapes, using lumped L and C, and multiple and long stages seen in others’ works, the new balun has single-stage hybrid coupler and stage- lines and phase-shifter, and complete distribute elements and its design is elaborated on in detail. The dipole antenna is different from others by having two arms in the form of hybrid metamaterial lines and have the same omni-directional pattern at the two resonance frequencies; 2.4 and 5.2 GHz are chosen as the test case and the circuit approach is done first, and followed by the EM simulation and the measurement. Additionally, the CRLH property is shown with the dispersion diagram and the size reduction effect of the proposed balun is addressed.

## 2. Compact CRLH Dual-Band Balun

### 2.1. CRLH Dual-Band Hybrid Branch-Line Coupler

In this section, the design of a dual-band balun as fully-printed distributed-elements without lumped elements is explained. The size of the entire structure will be much reduced by devising a CRLH phase shift-line to have 90° at the lower frequency band and −90° at the higher frequency band, and by forming a compact branch-line coupler. 

The circuit of the CRLH phase-shift line is set up by solving the equations to get the dual-band properties as the hybrid branch line coupler. Before starting the process of the design, as a goal, the following specifications in Table 1 are given as follows.

**Figure 1:**
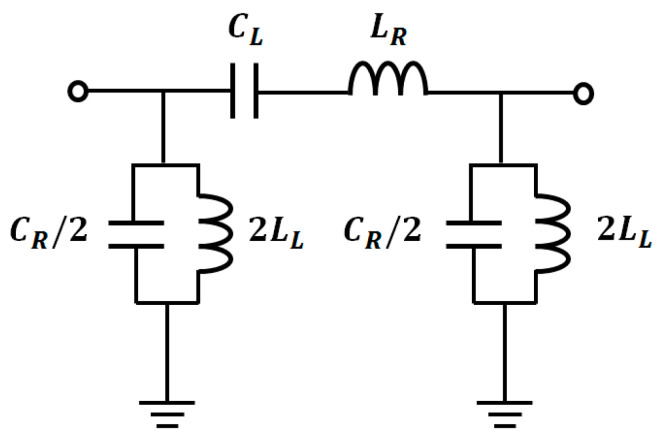
Equivalent circuit of the proposed dual-band CRLH phase-shifter line.

**Figure 2:**
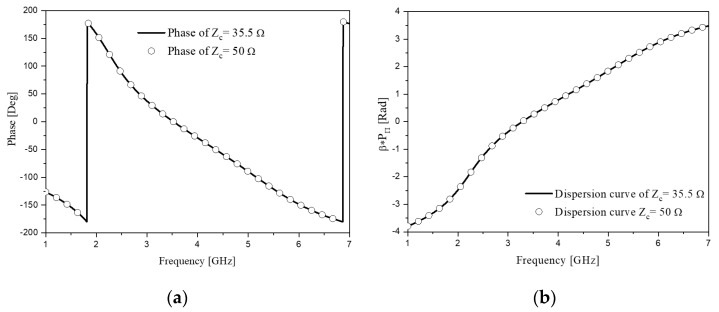
Circuit simulated results of the CRLH phase-shift line: (**a**) phase; (**b**) dispersion diagram.

In Figure 1, *C_R_*, *C_L_*, *L_R_* and *L_L_* are determined by generating 90° at *f*_1_, and −90° at *f*_2_. It is a matter of course, the zeroth-order resonance (ZOR) should also be created around (*f*_1_ + *f*_2_)/2 for the size-reduction. So, the mathematical equations for *C_R_*, *C_L_*, *L_R_* and *L_L_* are as follows [22,23]. Others end up with multiple stages, but a single-stage circuit is proposed here to reduce the size.

$$L_{R}=\frac{Z_{c}\pi [\left(\frac{\omega_{1}}{\omega_{2}}\right)+1)]}{{2\omega }_{2}[1-(\frac{\omega_{1}}{\omega_{2}}{)}^{2}]},\;C_{L}\;=\frac{\pi [\left(\frac{\omega_{1}}{\omega_{2}}\right)+1)]}{{2\omega }_{2}Z_{c}[1-(\frac{\omega_{1}}{\omega_{2}}{)}^{2}]}$$

$$L_{L}=\frac{{2Z}_{c}[1-\left(\frac{\omega_{1}}{\omega_{2}}\right))]}{{\pi \omega }_{1}[1+(\frac{\omega_{1}}{\omega_{2}}{)}^{2}]}{,\;C}_{R}=\frac{2\pi [\left(\frac{\omega_{1}}{\omega_{2}}\right)+1)]}{\omega_{1}Z_{c}[1+(\frac{\omega_{1}}{\omega_{2}}{)}^{2}]}$$

where $\omega_{1}=2\pi f_{1}$, $\omega_{2}=2\pi f_{2}$ and Z_c_ characteristic impedance in above equations. Solving the equations by setting Z_c_, *f*_1_ and *f*_2_ at 35.5 Ω, 2.4 and 5.2 GHz, *C_R_*, *C_L_*, *L_R_* and *L_L_* are as 2.52 pF, 0.64 pF, 3.17 and 0.81 nH, respectively. Additionally, to get the 50-Ω case, tackling the equations as *f*_1_ and *f*_2_ at 2.4 and 5.2 GHz the values are as follows: 1.78 and 0.46 pF, 4.46 and 1.14 nH, in the same order with the 35.5-Ω case. Using these elements, the phase and dispersion diagrams are plotted and show the CRLH characteristics including the ZOR point as in Figure 2.

In Figure 2a, the phases of 90° and −90° are achieved at 2.4 and 5.2 GHz. This phase-shift line complies with the specifications in Table 1 and will put into the 90°-branches of the hybrid branch-line coupler for miniaturization in terms of the physical size. Besides, there is the dispersion diagram in Figure 2b where the curve shows the LH region (β < 0) and the RH region (β > 0), along with the zeroth-order resonance (ZOR) near 3.8 GHz.

**Figure 3:**
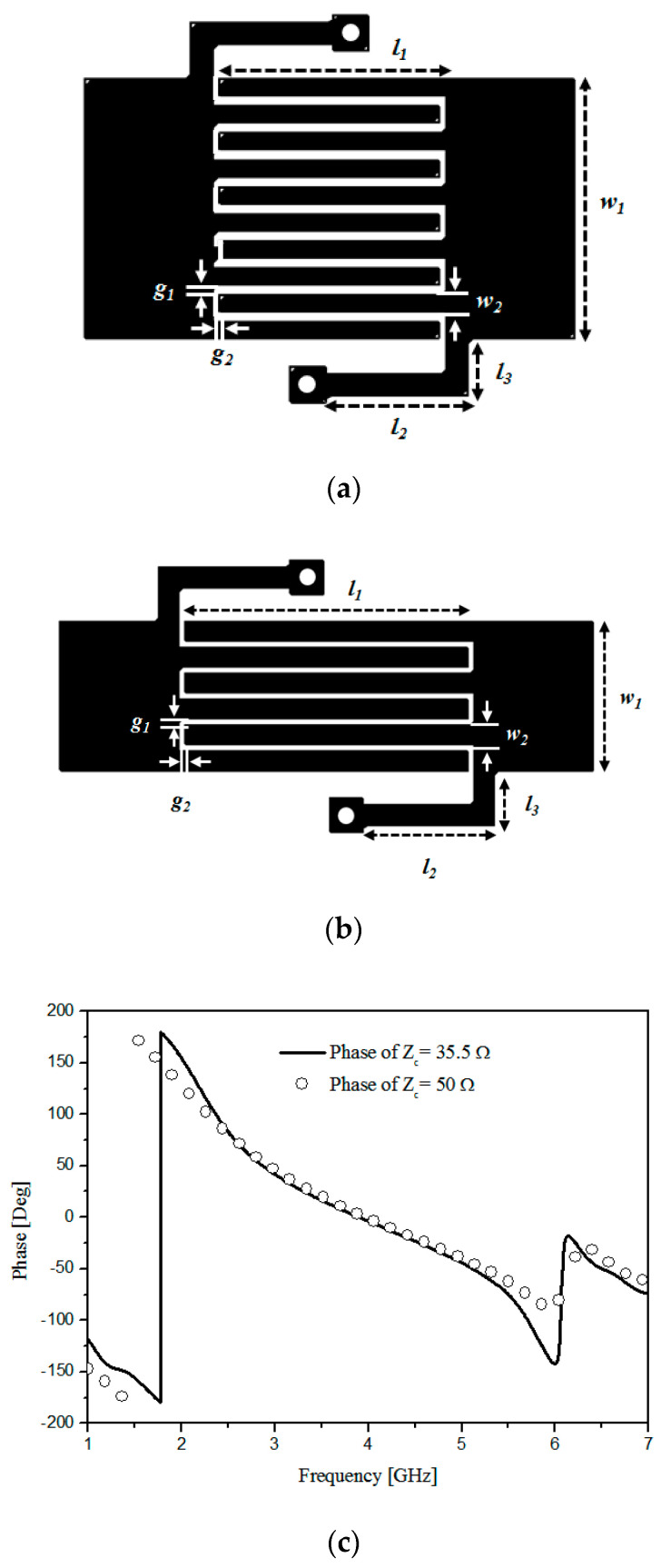
The physical geometries of the phase-shifter for (**a**) 35 Ω and (**b**) 50 Ω of their phases (**c**).

The finalized physical dimensions of the 35.5 Ω phase-shift line and geometry are given as table II and Figure 3a. Additionally, the physical dimensions of the 50 Ω phase-shift line and geometry are given as Table 2 and Figure 3b. The single-stage fully printable CRLH phase-shift line is designed using the CST-MWS as a full-wave EM simulator. Figure 3 shows the proposed CRLH phase-shift line geometry and EM simulated data of the phases. Both 35.5 and 50 Ω have an interdigital and shorting structure for C_R_, C_L_, L_R_ and L_L_. The phases of 90° and −90° are realized at 2.4 and 5.2 GHz at 35.5 and 50 Ω. These will be substituted for the 90°-branches of the hybrid-branch-line coupler with a view to the effective size reduction of the dual-band balun.

**Figure 4:**
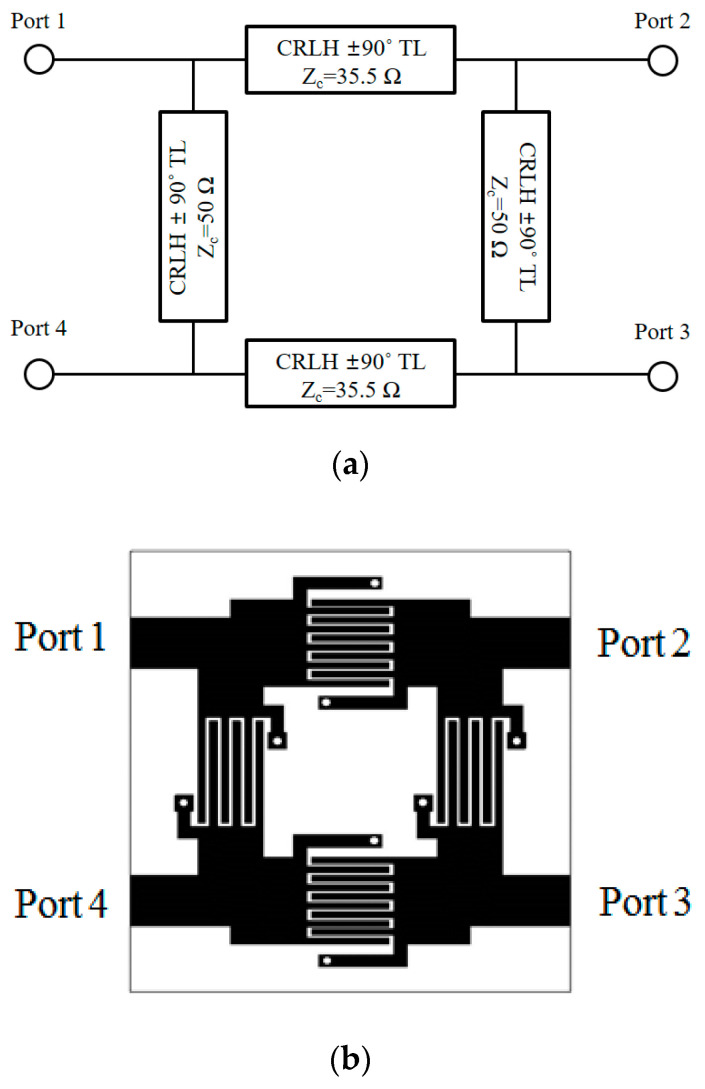

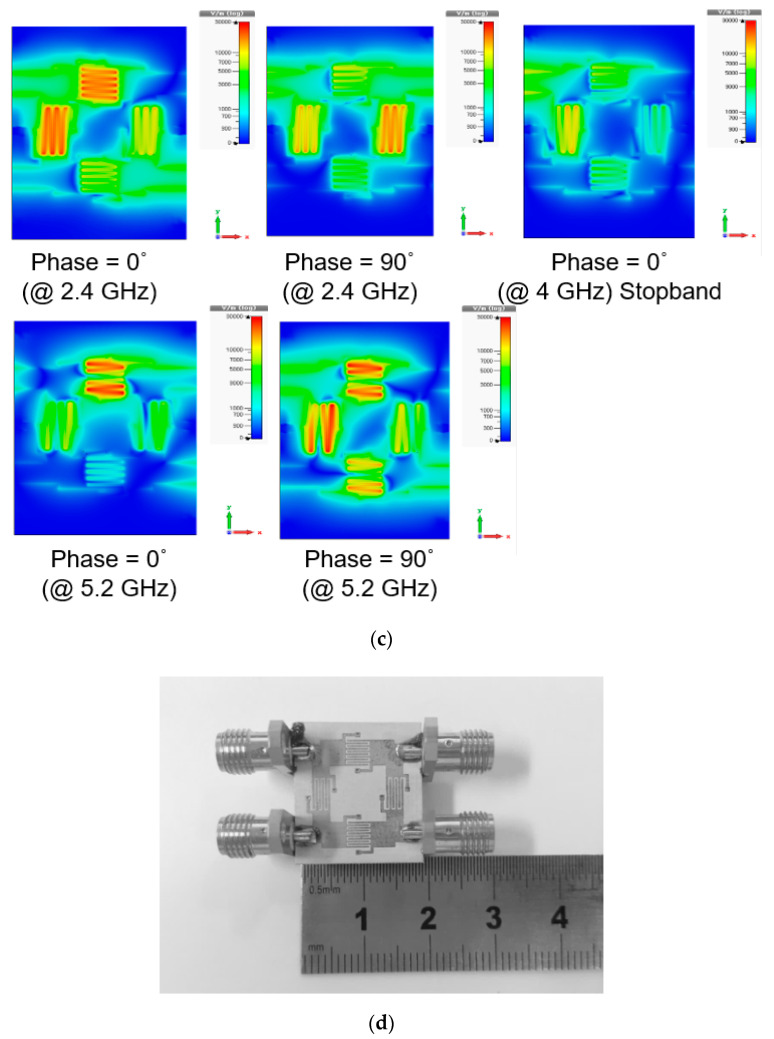
The physical geometry of the dual-band CRLH hybrid branch-line coupler as a single-stage geometry: (**a**) schematic; (**b**) EM (Electromagnetic) design; (**c**) E-field distribution; (**d**) fabricated prototype.

Each of the segments in Figure 4a is realized with Table 2 for Figure 3a,b and becomes Figure 4b. As a crucial building block of the proposed balun, the function of the dual-band branch-line coupler is checked with the electric-field distributions at the frequencies of interest as in Figure 4c. The RF energy from port 1 is split into the output ports, and port 2 is turned on first as the 0°- field-shot, and then port 3 is turned on after 90°-lapse for 2.4 and 5.2 GHz. Nonetheless, at 4 GHz as the stopband, the electric field is not detected at the output ports in the 0°-field-shot and 90°-shot. The geometry is physically realized as in Figure 4d where the length is about 2 cm.

**Figure 5:**
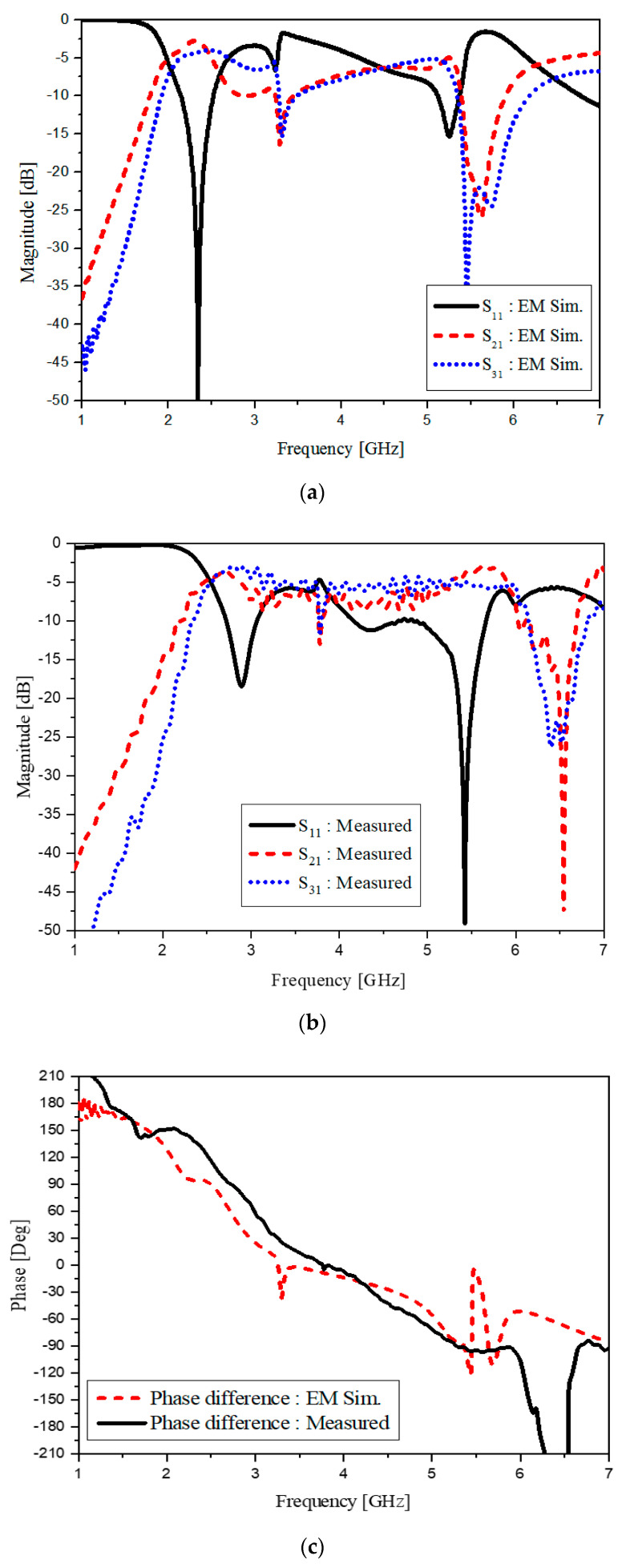
The frequency response of the dual-band CRLH hybrid branch-line coupler (**a**) EM simulated; (**b**) measured; (**c**) phase difference.

Figure 5a shows S_11_, S_21_ and S_31_ of the EM simulation From S_11_, the impedance is matched at the two frequencies. S_21_ and S_31_ present the equal power-division at the outputs. Figure 5b has the measured S_11_, S_21_ and S_31_. Similarly, to the simulated results, the power-division as well as the impedance match are obtained at 2.4 and 5.2 GHz. Figure 5c reveals the phase difference obtained as expected for the branch-line coupler. Somewhat incomplete parts of the function will be mitigated in the stage of the balun.

### 2.2. CRLH Dual-Band Balun

**Figure 6:**
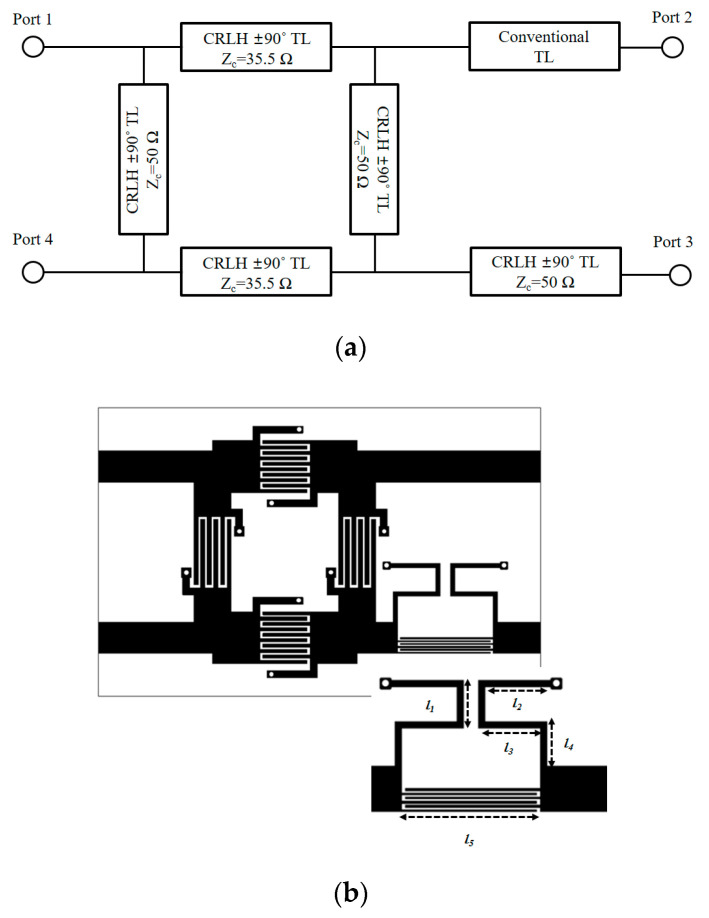

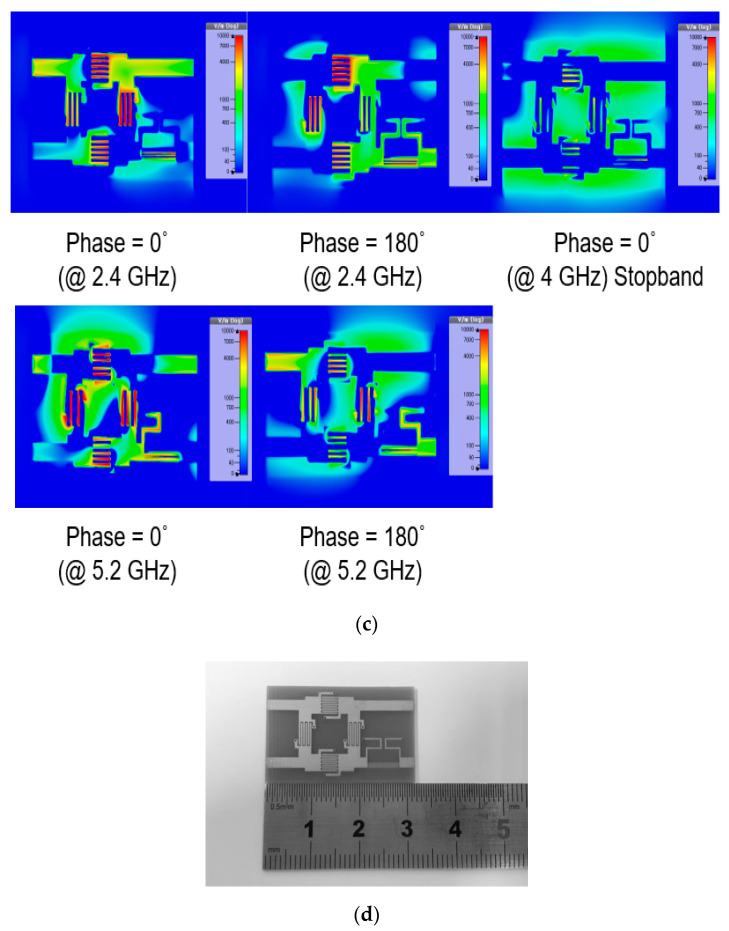
The physical geometry of the dual-band CRLH balun as a single-stage geometry: (**a**) schematic; (**b**) EM design; (**c**) E-field distribution; (**d**) fabricated design.

**Figure 7:**
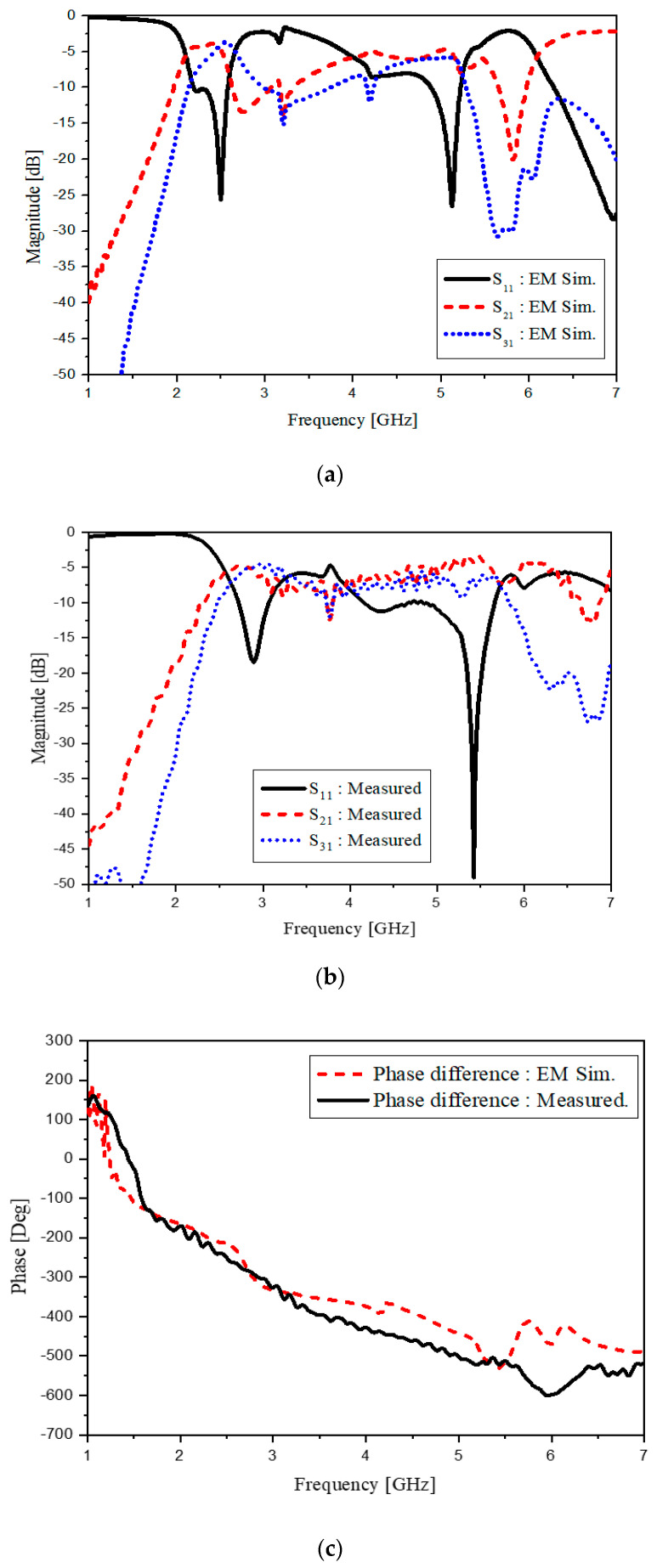
The frequency response of the dual-band CRLH balun: (**a**) EM simulated; (**b**) measured; (**c**) phase difference.

The branch-line coupler of Figure 4a is developed to the metamaterial balun of Figure 6a in the level of the schematic. The implementable geometry of the CRLH hybrid branch-line coupler extended with the CRLH phase-shift lines is shown in Figure 6b. Prior to fabrication, electromagnetic observation is conducted on the suggested device. The purpose of this observation is to see this device working as a desirable balun that shows the 180°-phase difference between ports 2 and 3 from the standpoint of the electric field. In Figure 6c, for 2.4 and 5.2 GHz, the RF energy is divided to ports 2 and 3, and port 2 and port 3 take turns by a 180°-time lapse. Meanwhile, at 4 GHz, bot output ports appear dark, which means no energy passes the circuit. As to the fabricated balun, its overall size is 14 × 20 × 1.2 mm^3^ and realized on the FR4 consistent to the former design stage. Considering 27 × 27 × 1.2 mm^3^ as the nominal size of other dual-band baluns with FR4, the size is reduced more than four times. The proposed balun shows similar results to circuit design as shown in Figure 7. The dual-band operating frequency is 2.4 and 5.2 GHz as satisfying the specifications in Table 1. The curves of S_21_ and S_31_ are almost the same each other as the −3.8 dB. This also results from the use of FR4 substrate. Here comes 180° as the phase difference at *f_1_* and *f_2_*.

The frequency response of this compact balun works as expected. In Figure 7, both the simulated and measured s-parameters meet the design specifications. Additionally, the simulation and measurement results are in good agreement. The 180°-phase difference is seen. With regard to the size of the dual-band balun from the dual-band hybrid branch-line coupler, there is no change even though there is one new component added to the hybrid branch-line coupler. Since the new component as the dual-band phase-shift line is made as part of output port 3, it does not work as something negative in the size-reduction. Next, a dual-band dipole printable on the substrate is elaborated on.

## 3. CRLH Dual-Band Dipole

**Figure 8:**
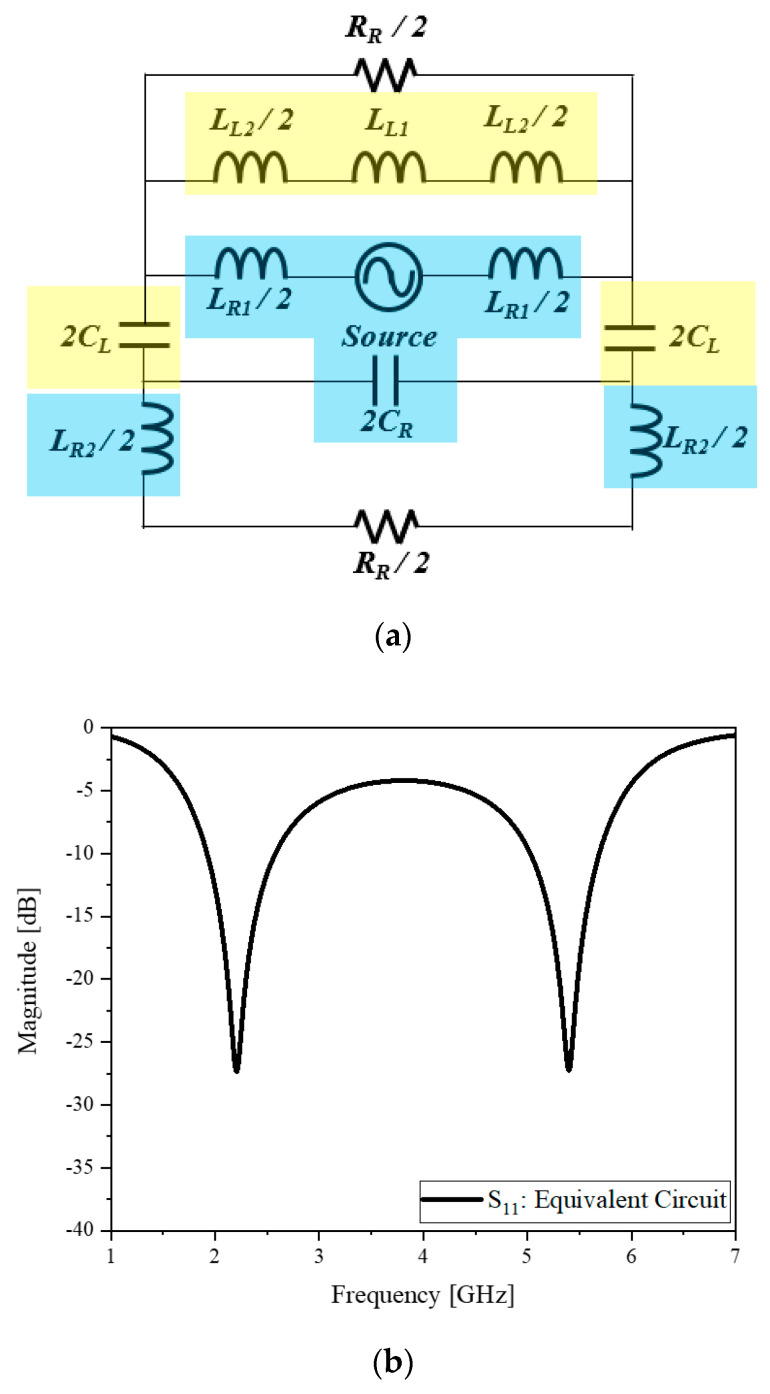

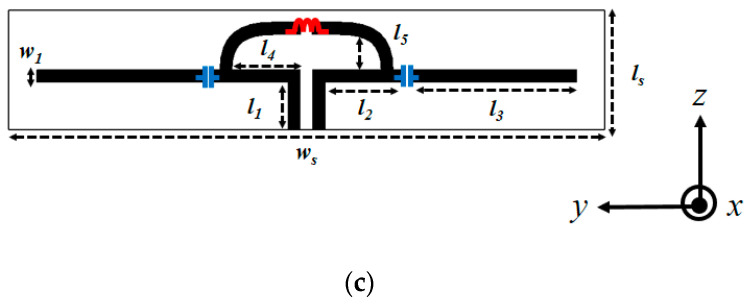
The CRLH dual-band dipole antenna design: (**a**) equivalent circuit; (**b**) S_11_ of the equivalent circuit; (**c**) structure in the EM CAD (Computer aided design) program.

The motivation of this part of the work is to make a dipole resonate at two frequencies not in the harmonic relationship and have the same omni-directional pattern at different resonance frequencies as a dipole. The proposed CRLH dipole antenna which has a dual-band property and operating at 2.4 and 5.2 GHz shown in Figure 8. In Figure 8a the highlighted cyan section indicates the upper path and yellow section express the lower path as an equivalent circuit for Figure 8b where R_R_ = 67 Ω, L_R1_ = 3.9 nH, L_R2_ = 25.5 nH, L_L1_ = 12 nH, L_L2_ = 2 nH, C_R_ = 0.38 pF, C_L_ = 0.5 pF. These result in the resonance at 2.4 and 5.2 GHz as in Figure 8c. In contrast to other dual-band dipoles, to maintain the omni-directional radiation pattern by suggesting a bow shape structure, a new structure is designed by adding a new path and making the entire geometry as the hybrid metamaterial arms on FR4 substrate. The upper current path is used to excite the high frequency and lower side path is matching at the low frequency. So, the proposed antenna connected from the top as the lumped inductor through the bottom path lumped capacitors as a loop shape. Geometrical elements w_s_, w_f_, w_1_, w_2_, w_g_, g_1_, g_2_, l_s_, l_f_, l_1_, l_2_, l_3_, l_4_, and l_g_ are 100, 3, 80, 3, 150, 5, 150, 85, 20, 22, 15, 10, 85, and 3, respectively in mm. Compared to 140 mm or larger in length for commercial dipoles which are volumetric with the dielectric enclosure, the proposed antenna is smaller and planar. Using the geometrical parameters, the simulated and measured S_11_ as well as the far-field pattern is provided next.

**Figure 9:**
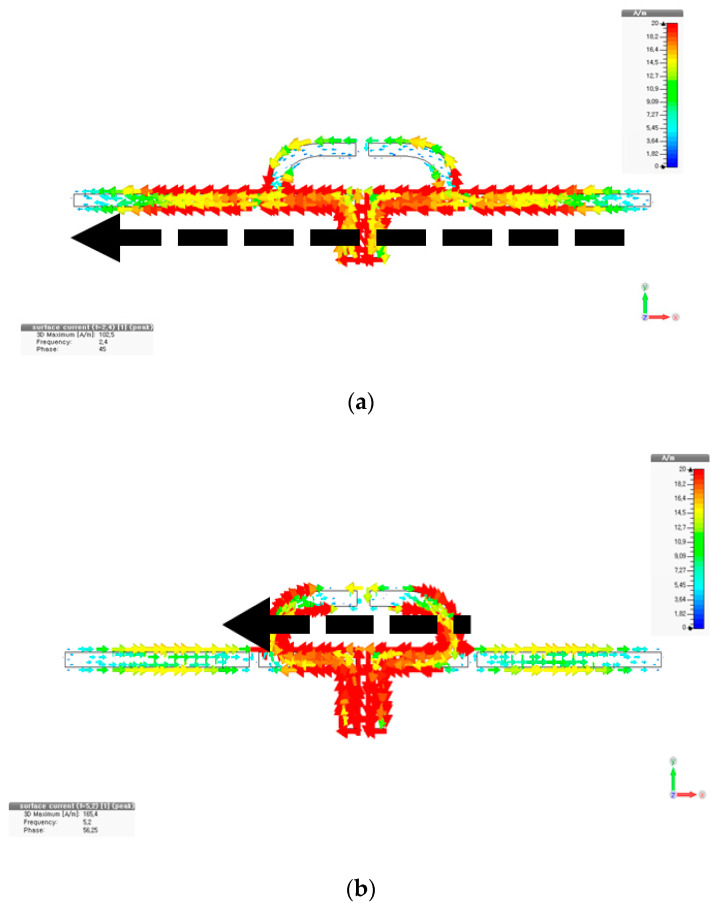

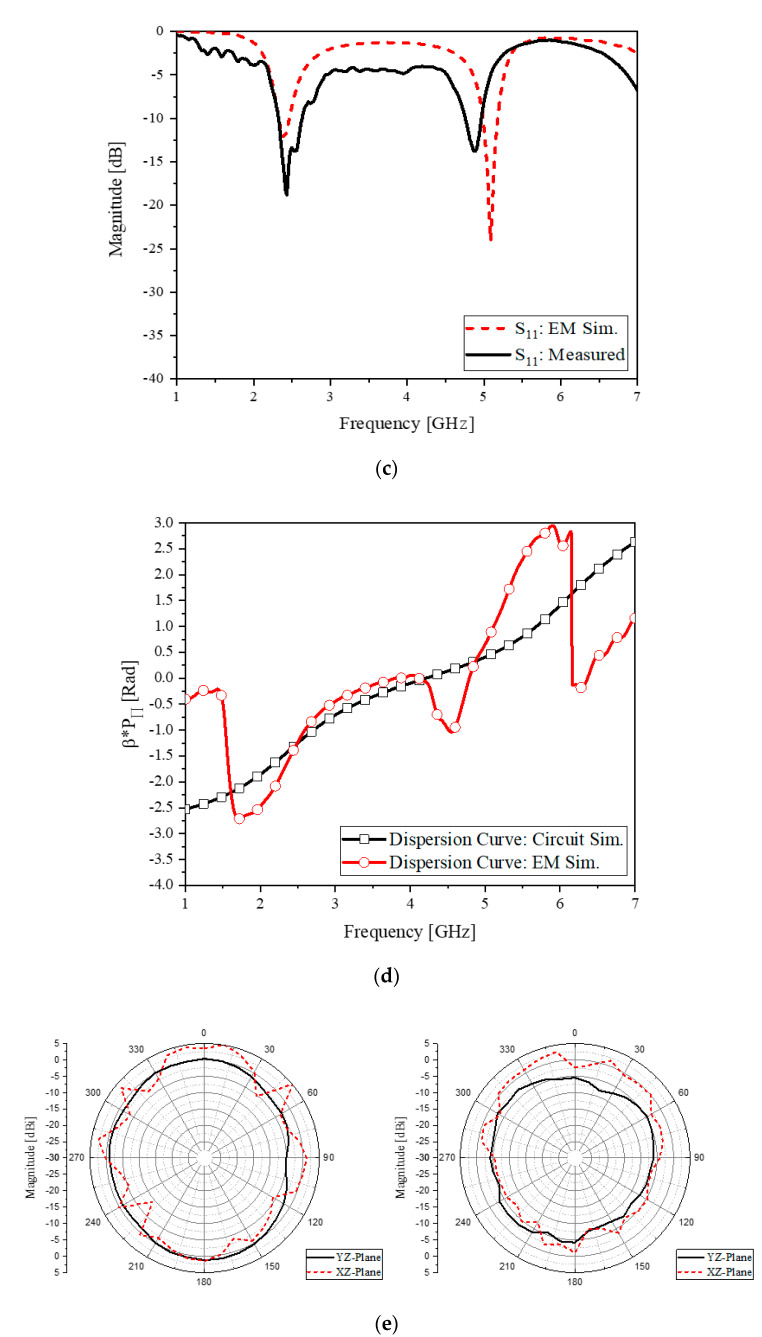
CRLH dual-band dipole: (**a**) current at 2.4 GHz; (**b**) current at 5.2 GHz; (**c**) S_11;_ (**d**) dispersion curves; (**e**) measured beam-patterns.

Figure 9a,b expresses the resonant currents. The EM simulated and measured S_11_ in Figure 9c agree well and comply with the objectives. Dispersion curves are obtained from the circuit model and EM model of the new dipole as in Figure 9c. While 5.2 GHz has a positive wavenumber, 2.4 GHz has a negative wavenumber, which reveals the metamaterial property, helpful to size-reduction. Additionally, the far-field patterns with antenna gains 3.9 dBi and 2.38 dBi for the two frequencies seem very similar as given in Figure 9e.

## 4. CRLH Dual-Band Antenna with CRLH Dual-Band Balun

**Figure 10:**
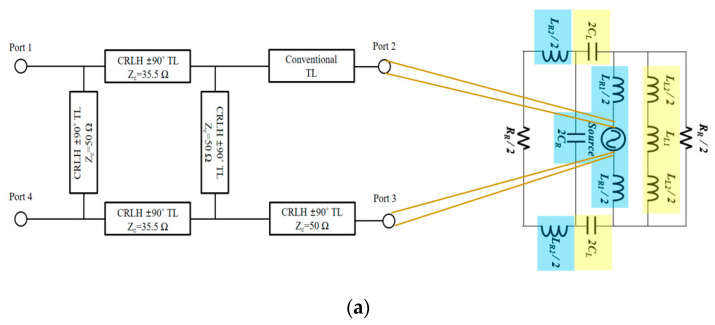

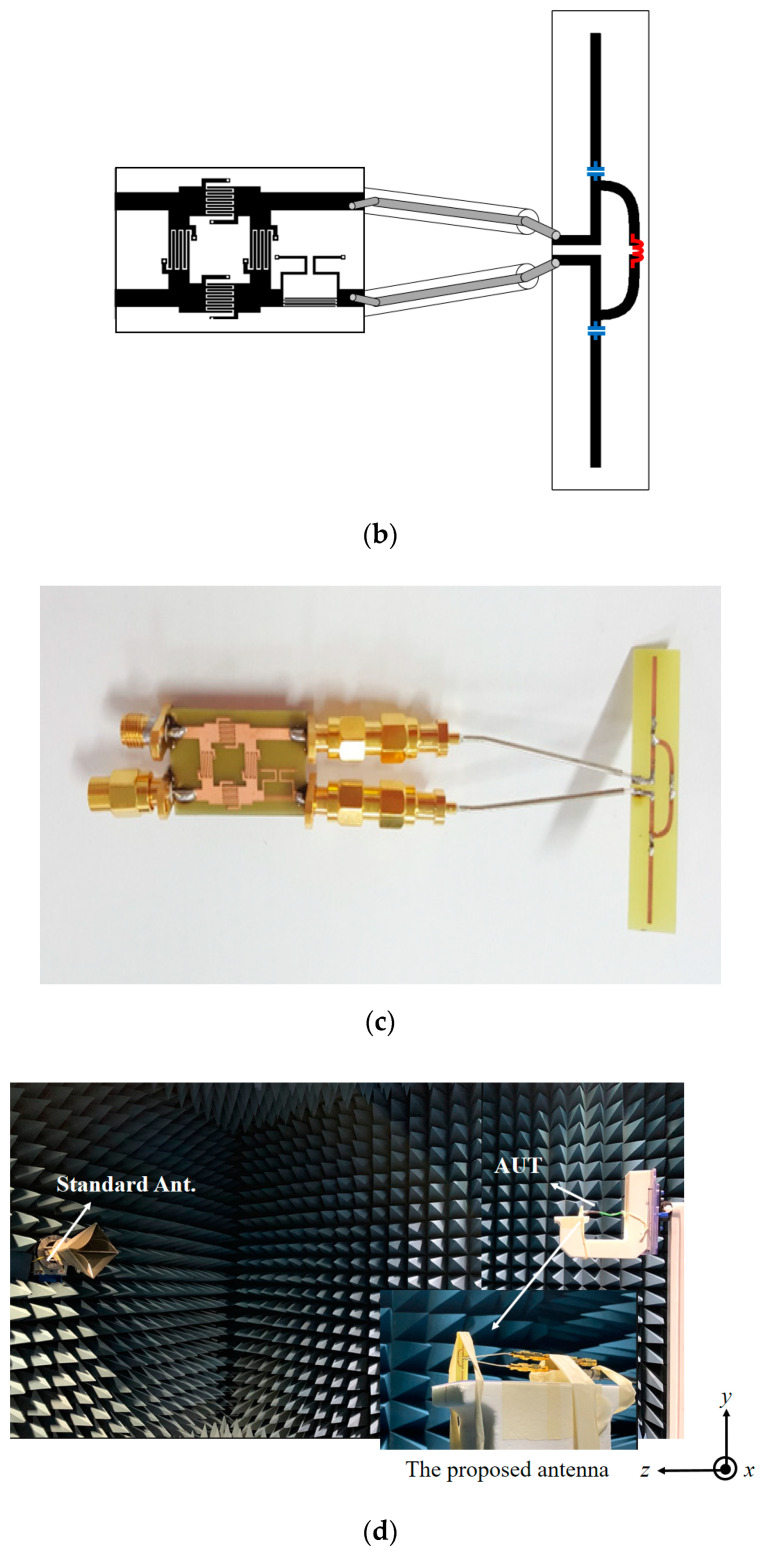
The CRLH dual-band dipole fed by the CRLH balun: (**a**) schematic; (**b**) hybrid schematic; (**c**) fabricated geometry; (**d**) far-field pattern measurement facility.

**Figure 11:**
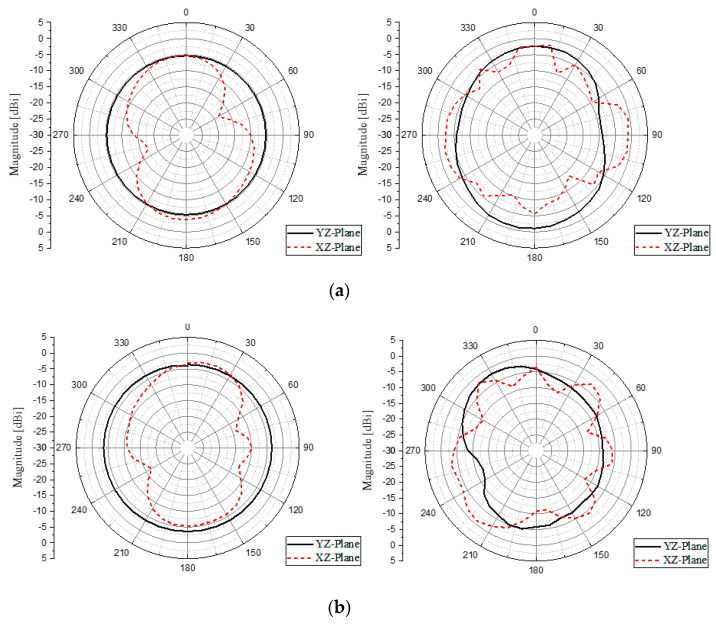
The radiation patterns of the CRLH dual-band dipole with CRLH balun: (**a**) simulated and measured results at 2.4 GHz; (**b**) simulated and measured results at 5.2 GHz.

Figure 10a is a sketch of the idea how the balun is used to feed the circuit model of the dipole antenna. The realized CRLH dual-band balun and antenna structure are provided in Figure 10b,c. The measurement harness at an anechoic chamber is seen in Figure 10d. The antenna gains are observed and they are above −1 dBi at the two frequencies. Table 3 presents the simulated and measured antenna gains. Figure 11a,b shows the gains and radiation characteristics of the CRLH dual-band antenna at 2.4 and 5.2 GHz are obtained similar to Figure 9. As to the antenna efficiency, it is over and similar to 50% in simulation, and becomes lower than 40% in measurement as checked in Table 3. They are changed from the simulated results in a small scale, due to the manual soldering for wiring of the semi-rigid coaxial cables and connector adapter as a major reason as well as the deviation of FR4 dielectric constant from the vendor’s data. For example, it was found out the inductor was soldered in the lab which has the effect of adding an extra inductance and pushes the second resonance frequency downward by approximately 0.4 GHz. The fabricated antenna gives a not-very-high efficiency due to the aforementioned errors, but the efficiency at the two frequencies is usable for communication as guessed from practices. With regard to the approach of combining the dual-band balun with the dual-band dipole, the gain of the antenna falls by 0.7 dB at 2.4 GHz and 1.1 dB at 5.2 GHz in the simulation because of adding the balun. This is loss, but the antenna gain is nearly 1 dBi. The loss grows in the measurement as 5 dB at 2.4 GHz and 4 dB at 5.2 GHz, even though the antenna gain for the two frequencies is below 0 dBi. The loss from the combination is accounted for by the 1-dB insertion loss of the manufactured balun, and the 3.5 dB of the mismatch from cabling and connector attachment. Precision etching with a low-loss substrate and cascading all the blocks without the cables will mitigate this loss problem. As for the far-field patterns of the antenna is broad and close to the omni-directional shape. This can be an obvious advantage, as a small planar balun feed and a planar dipole enabling the same radiation function working at different frequencies, while commercial baluns and dual-band dipoles are bulky and give difficulty in being integrated to flat wireless systems for diverse use-cases such as IoT (Internet of Things) apparatus.

## 5. Discussion

It is meaningful to check the technical status of the proposed idea in reflection of others’ in the same subject. First, comparison is made between the new balun and other baluns aiming at multiple bands.

Through Table 4, the features of the new metamaterial balun are mentioned with those of selected papers as above. The kinds of features are type of structure, frequency bands, size, metamaterial or not, and origin of the design. While Tseng et al proposed the mixture of distributed and lumped elements, this work and the others are made from distributed elements, which provides ease of manufacture [16]. Except for this report, the circuits work for 2.4 and 5.2 GHz, and both works adopt the concept of metamaterials. They are obviously different in the following observations Tseng [16] uses multiple stages of lumped elements as a metamaterial line and a conventional delay-line to be a power-divider not a hybrid coupler. However, the proposed structure has no lumped elements and single-stage metamaterial-line segments for a hybrid coupler not a power-divider. Size-wise, the proposed balun is much smaller than others’. Huang, F et al presented a little bit smaller than the proposed geometry because their line is diagonally folded into the center of the area and the input port is connected to the common corner, but this work has not folded the branch-line coupler. The other circuits originate from the power-divider, filter, and T-junction, but the backbone of this work is the branch-line coupler similar to Yang et al’s design [18]. Please note, Yang’s device [18] is not a metamaterial but a coupler with stubs. This comparison is convincing that the proposed balun is novel and appropriate for the objectives. Second, it is worth checking characteristics and benefits of the proposed dual-band dipole in comparison with others in the same subject.

The antennas are looked into in terms of type, frequency, size, whether metamaterial or not, layering, and with or without a balun as in Table 5. Deng, Li, and Deepak’s strucutres [1,2] and [6] are PIFA, bow-tie and SIR, respectively, but most of them take the form of a dipole or its modification. In contrast to all, only this work uses a metamaterial dipole. While Li, Huang, Sim, and Azeez’s antennas [2,4,7] and [10] have more than three bands, 2.4 and 5.2 GHz are obtained by the other dipoles. To check the sizes as miniaturization effect, the areas of all the antennas are compared, which leads to a finding that the proposed antenna is substantially small. As given a question which antennas are metamaterials, this work (with Huang and Pushpakaran’s antennas [4] and [5]) answers the question. Huang and Pushpakaran’s antennas [4] and [5] are patch with a slit and slit coupled patches, respectively, and they have no evidence of metamaterials. However, this work reveals metamaterial properties with Figure 2b and Figure 9d. With regard to layering, which is related to cost, Li, Huang, Pushpakaran, Huang, and Alekseytsev’s antennas [2,4,5,8] and [11] have two or three layers. Nonetheless, this work is realized with a single layer of dielectric, which is cost effective. Most of them do not have baluns, but Huang et al’s antenna [8] uses a 2-faceted balun comprising an upper tapered line and a lower line, and this work is connected with the CRLH balun of a single layer. The proposed antenna and CRLH balun have distinct features as explained.

## 6. Conclusions

A dual-band balun and a dual-band dipole antenna for the 2.4 and 5.2 GHz wireless communication are designed and combined to meet the demands on higher throughputs in IoT mobile connectivity with standing and portable electronic products. These two blocks are novel and small in that different from others, distributed type and single-stages of CRLH TX-lines are basically used to meet the requirement on the dual-band performance. In detail, the balun originates from a branch-line coupler and its segments which are 90°-lines in the conventional designs are replaced by metamaterial phase-shifters that show −90°-phase at 2.4 GHz and +90°-phase at 5.2 GHz as a small structure. The one-stage compact balun eventually makes the output ports out of phase by 180° at the target frequencies. In order to coincide with the two frequencies of the balun, an ordinary dipole is changed to a CRLH structure where a negative phase and a positive phase are generated at 2.4 and 5.2 GHz as the resonance condition of the dipole. The size does not grow, since the negative phase occurs at the lower frequency. The two bands of the dipole are fed by the balanced currents from the CRLH balun. This enables the cascaded blocks to radiate the far-field wave omni-directionally at the two frequencies with the acceptable antenna gains greater than −1 dBi, despite the error in the experiment due to the manual soldering of cabling as a major reason and the dielectric constant deviating from the vendor’s data as a minor one.

## Figures and Tables

**Table 1:** The specifications of the composite right- and left-handed (CRLH) dual-band balun.

Spec. Items	Feature	
Band	*f_1_*	2.4 GHz
	*f_2_*	5.2 GHz
Insertion Loss	Hybrid	<1.5 dB
	Balun	<1.5 dB
Return Loss	<−15 dB	
Isolation	<−15 dB	

**Table 2:** Physical dimensions of the proposed CRLH phase-shift line.

Items (35.5 Ω)	*l_1_*	*l_2_*	*l_3_*	*w_1_*	*w_2_*	*g_1_*	*g_2_*
Value (mm)	3.2	2	0.8	3.65	0.23	0.1	0.1
Items (50 Ω)	*l_1_*	*l_2_*	*l_3_*	*w_1_*	*w_2_*	*g_1_*	*g_2_*
Value (mm)	4.4	2	0.8	2.18	0.28	0.1	0.1

**Table 3:** Antenna gain and efficiency.

Type	Frequency	Sim./Meas.	Peak gain	Eff.
w/o Balun	2.4 GHz	Sim.	1.64 dBi	91%
		Meas.	5.13 dBi	88%
	5.2 GHz	Sim.	2.23 dBi	65%
		Meas.	2.93 dBi	47.9%
w/Balun	2.4 GHz	Sim.	0.9 dBi	35%
		Meas.	−0.48 dBi	21%
	5.2 GHz	Sim.	1.1 dBi	30%
		Meas.	−0.96 dBi	27%

**Table 4:** Comparison of the proposed CRLH dual-band balun and the previous works.

**Ref.**	**Type**	**Freq.**	**Size**	**Metamaterial**	**Origin**
[16]	Distributed & Lumped (Hybrid)	1.24–3.58 GHz	0.40 λ_0_ × 0.53 λ_0_	o	Power divider
[17]	Distributed	2.45/5.2 GHz	0.3 λ_0_ × 0.19 λ_0_	x	Bandpass filter
[18]	Distributed	2.45/5.25 GHz	0.7 λ_0_ × 0.32 λ_0_	x	Hybrid coupler
[19]	Distributed	2.4/5.2 GHz	0.15 λ_0_ × 0.15 λ_0_	x	T-junction
[20]	Distributed	2.4/5.2 GHz	0.65 λ_0_ × 0.65 λ_0_	x	T-junction
This work	Distributed	2.4/5.2 GHz	0.24 λ_0_ × 0.16 λ_0_	o	Hybrid coupler

**Table 5:** Comparison of the proposed CRLH dual-band dipole antenna and the previous works.

Ref.	Type	Frequency	Size	Metamaterial	Layer	Balun
[1]	PIFA	2.4/5.2 GHz	0.15 λ_0_ × 0.1 λ_0_	x	Single	X
[2]	Bow-tie	2.4/3/5.2 GHz	0.4 λ_0_ × 0.36 λ_0_	x	Double	X
[4]	Monopole	2.4/3.5/5.2~5.8 GHz	0.05 λ_0_ ×1 λ_0_	o	Double	X
[5]	Dipole	2.4/5.2 GHz	0.3 λ_0_ × 0.32 λ_0_	o	Triple	Slot line transition
[6]	SIR	2.4/5.2 GHz	0.22 λ_0_ × 0.16 λ_0_	x	Single	X
[7]	Asymmetric Dipole	2.4/5.2/5.8 GHz	0.25 λ_0_ × 0.05 λ_0_	x	Single	X
[8]	Dipole	2.4/5.2 GHz	0.6 λ_0_ × 0.4 λ_0_	x	Double	2 faceted-balun
[9]	Dipole	2.4/5.2 GHz	0.2 λ_0_ × 0.1 λ_0_	x	Single	X
[10]	E-shape Dipole	2.4/5.2/5.8 GHz	0.65 λ_0_ × 0.65 λ_0_	x	Single	X
[11]	Quasi-Yagi	2.0/2.4 GHz	1.17 λ_0_ × 0.56 λ_0_	x	Double	Slot line transition
This work	CRLH Dipole	2.4/5.2 GHz	0.4 λ_0_ × 0.08 λ_0_	o	Single	○
